# The Role of Central Neck Lymph Node Dissection in the Management of Papillary Thyroid Cancer

**DOI:** 10.3389/fonc.2017.00122

**Published:** 2017-06-19

**Authors:** Lawrence A. Shirley, Natalie B. Jones, John E. Phay

**Affiliations:** ^1^Department of Surgery, Division of Surgical Oncology, Ohio State University Medical Center, Columbus, OH, United States; ^2^Ohio Health, Columbus, OH, United States

**Keywords:** papillary thyroid cancer, central neck dissection, lymph nodes, prophylactic surgery, surgical complications

## Abstract

Papillary thyroid cancer (PTC) is the most common thyroid malignancy, and cervical nodal metastases are frequent at presentation. The most common site for nodal metastases from PTC is the central compartment of the ipsilateral neck in the paratracheal and pretracheal regions. The decision to resect these lymph nodes at the time of thyroidectomy often depends on if nodes with suspected malignancy can be identified preoperatively. If nodal spread to the central neck nodes is known, then the consensus is to remove all nodes in this area. However, there remains significant controversy regarding the utility of removing central neck lymph nodes for prophylactic reasons. Herein, we review the potential utility of central neck lymph node dissection as well as the risks of performing this procedure. As well, we review the potential of molecular testing to stratify patients who would most benefit from this procedure. We advocate a selective approach in which patients undergo clinical neck examination coupled with ultrasound to detect any concerning lymph nodes that warrant additional evaluation with either fine needle aspiration or excisional biopsy in the operating room. In lieu of clinical lymphadenopathy, we suggest the use of patient and disease characteristics as identified by multiple groups, such as the American Thyroid Association and European Society of Endocrine Surgeons, which include extremes of ages, large primary tumor size, and male gender, when deciding to perform central neck lymph node dissection. Patients should be educated on the potential long-terms risks versus the lack of known long-term benefits.

## Introduction

Papillary thyroid cancer (PTC) makes up approximately 74–85% of all forms of thyroid cancer. The incidence of PTC has been rising in the United States over the past 30 years from 3.6 per 100,000 in 1973 to 9.1 per 100,000 (females) and 2.9 per 100,000 (males) in 2011 (1). Most of this increase in PTC can be attributed to the incidental finding of small tumors (<1 cm) as a result of the increased use of ultrasound and computed tomography scans for a variety of other conditions. PTC is more common in women, whereas in men it tends to be more aggressive and occurs at a later age. Patients with PTC tend to have an excellent prognosis with an overall 10-year survival of 93% (2). Approximately 1,500 patients die per year of PTC and the patients who do succumb to the disease frequently develop respiratory failure due to extensive pulmonary metastases (3).

Lymph node metastases are a common occurrence in PTC, despite excellent long-term survival. Studies have demonstrated that micrometastases can be found in the central cervical lymph nodes in 40–60% of cases (4, 5). Preoperatively, patients should undergo a clinical neck examination in combination with a high resolution neck ultrasound in order to detect any concerning lymph nodes that would warrant further investigation. Unfortunately, lymph nodes in the central neck compartment are more difficult to image *via* ultrasound when compared to the lateral neck due to their proximity to the thyroid gland and air-filled trachea (5, 6). Ahn et al. found that the sensitivity of detection of lateral compartment lymph nodes was 94%, compared to 53–55% in the central neck (7). Concerning lymph nodes visualized on ultrasound should then be sampled using fine needle aspiration (FNA) if it will change the extent of the operation. Cytologic analysis should be performed, and thyroglobulin (Tg) washings may be done, although Tg washings may be prone to a high false-positive rate if the thyroid gland is still present. A finding of malignancy by FNA would lead to the removal of lymph nodes within the central compartment of the neck, so-called therapeutic central neck lymph node dissection (CLND) at the time of total thyroidectomy. A lymph node found to be highly suspicious by ultrasound criteria, which typically includes a cystic component or hyperechoic punctations, does not always need confirmation by FNA especially when characterized by an experienced ultrasonographer (8). Other less-specific ultrasound characteristics, including a round shaped and loss of a hilum, may raise enough suspicion that FNA may be beneficial. If, at the time of thyroidectomy, firm, enlarged, or discolored lymph nodes are identified, the decision can be made to complete a CLND to clear all potential lymph node metastases. Patients with coexisting chronic lymphocytic thyroiditis often have multiple enlarged benign inflamed lymph nodes, making the decision to perform a CLND difficult. Frozen section analysis of these nodes may aid in the decision about completing a CLND. However, when preoperative or intraoperative investigations do not demonstrate evidence of lymph node spread, the surgeon must then make the difficult decision of performing a CLND for prophylactic reasons.

## Definition of a Central Neck Lymph Node Dissection

**Figure 1:**
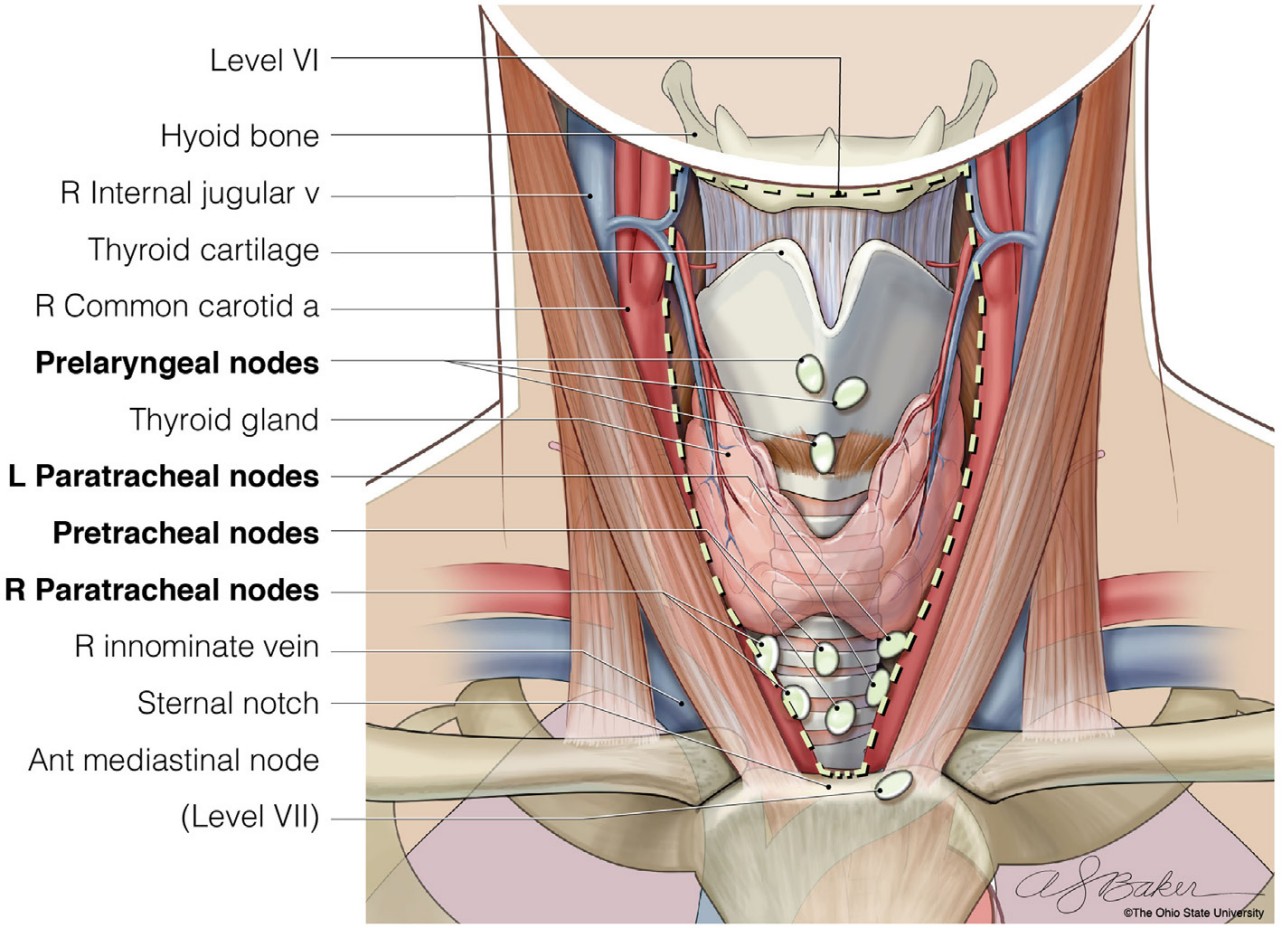
The anatomic borders of Level VI of the central neck (hyoid bone, carotid arteries, and sternal notch), where lymph nodes are resected when completing a bilateral central neck dissection. © Ohio State University.

The central neck compartment includes Level VI, the anatomic area bounded by the hyoid bone superiorly, the sternal notch inferiorly, and the medial borders of the carotid sheaths laterally (Figure 1). The structures found in this compartment are the esophagus, recurrent laryngeal nerves, trachea, parathyroid glands, thymus, and thyroid gland. The lymph nodes included in this compartment are the paratracheal, prelaryngeal, and pretracheal nodes. Lymph nodes found from the level of the sternal notch to the level of the innominate vein are described as Level VII lymph nodes, some or all of which are often removed during CLND (9, 10). Within the central compartment, lymph nodes tend to be more abundant superior to the isthmus (also called Delphian nodes) and inferior to the lobes bilaterally with fewer located above the superior poles of the thyroid. An ipsilateral central neck dissection involves removal of nodes on the same side as the thyroid cancer, whereas a bilateral CLND would include resection of all lymph nodes found in this central compartment. As the inferior thyroid artery commonly supplies both parathyroid glands, care must be taken not to injure this vessel during dissection. The recurrent laryngeal nerves should be directly visualized throughout the nodal dissection in order to avoid injury.

## Benefits and Risks of Prophylactic Central Neck Lymph Node Dissection

Unlike in virtually all other solid malignancies, initial studies on the prognosis of differentiated thyroid cancer failed to show prognostic significance of regional lymph node involvement. This was reflected in that traditional staging systems, including metastases, age, completeness of resection, invasion, size and age, metastases, extent of primary cancer, size, does not include lymph node status in determining prognosis. However, with the use of a larger database, Mazzaferri and Jhiang demonstrated that patients with lymph node metastases did, in fact, have a worse prognosis (11). Subsequent large-scale studies have generally corroborated these findings (12, 13), especially, in older patients. This is reflected in the current American Joint Committee on Cancer staging system, where lymph node involvement upstages patients older than 45 years from stage II to stage III. As it has taken large databases to show small but usually significant differences in survival with nodal disease, there remains a lack of good evidence that removing involved nodes improves survival. As prophylactic lymph node dissection by definition removes microscopic disease, it is generally accepted that this procedure does not change overall prognosis but may affect recurrence rates (14). However, convincingly demonstrating a recurrence difference may be challenging. The American Thyroid Association (ATA) assessed the feasibility of performing a randomized controlled trial to evaluate the benefit of prophylactic CLND and estimated that it would take over 5,000 enrollees and over $15 Million to perform (15). In general, the goals of prophylactic CLND are not to improve survival, but to potentially avoid reoperative high-risk surgery from recurrences, minimizing biochemical evidence of disease, and simplifying follow-up.

The decision to perform therapeutic CLND is dependent on information gained through clinical examination, preoperative ultrasound, and intraoperative assessment. However, these tools have been shown to be unreliable in determining the presence of microscopic lymph node metastases (16, 17). With this in mind, along with the known very high prevalence of central neck lymph node metastases, several groups advocate routine prophylactic CLND. Prophylactic CLND potentially decreases recurrent central neck disease. Hall et al. reported no central neck lymph node recurrences in 266 patients undergoing routine CLND (18). The potential oncologic benefit of routine CLND has been supported through studies showing that performing CLND was associated with decreased Tg levels postoperatively (19–21). Lower Tg levels would potentially increase the sensitivity for using Tg for long-term surveillance. As well, having undetectable Tg levels decreases patient’s mental anguish and could decrease surveillance frequency and cost. Lower Tg levels after CLND implies that removing central neck lymph nodes routinely reduces the burden of disease. A recent meta-analysis of 2,318 patients saw a trend toward lower recurrence rate when prophylactic CLND was performed, whereas this did not reach statistical significance (22). As previously mentioned, only an extremely large randomized control trial may definitively answer this question, but is unlikely to be performed.

Another potential benefit of prophylactic CLND has been to more accurately stage patients, which can clearly affect RAI treatment. In two retrospective studies, approximately one-third of patients older than 45 years were upstaged to stage III disease (23, 24). However, in a recently published randomized control trial (25), where 181 patients were randomized to total thyroidectomy plus prophylactic CLND versus total thyroidectomy alone, clinically relevant up-staging occurred in only one patient. Still, in patients with CLND, there was a reduced need to perform repeated I-131 treatments. Prophylactic CLND resulting in different radioactive I-131 ablation treatments has also been demonstrated in other retrospective reviews (26, 27). With the current ATA guidelines recommending assessing the size and number of lymph nodes involved before deciding on RAI treatment, prophylactic CLND may play an even larger role in determining RAI use. A prophylactic CLND that demonstrates a lack of lymph node metastasis would strengthen the case not to use RAI treatment in a low-risk patient. Thus, the performance of CLND can potentially have a significant impact on further treatment regimens, although the degree of impact remains a matter of debate.

CLND has several associated risks, including increased risk of hypoparathyroidism and recurrent laryngeal nerve injury over thyroidectomy alone (28). A meta-analysis of over 3,000 patients found that temporary hypocalcemia and temporary vocal cord paralysis were more common in patients undergoing CLND versus thyroidectomy alone but that there were no differences in permanent hypocalcemia or vocal cord injury (23). An Italian series of 1,087 patients assessed differences in complications with thyroidectomy plus bilateral CLND compared to ipsilateral CLND or no dissection. The bilateral CLND group had a significantly higher rate of permanent hypoparathyroidism compared to the other two groups (bilateral 16%, ipsilateral 7%, and no CLND 6.3%). There were no significant differences in the rates of permanent recurrent nerve injury although the bilateral group trended toward an increased injury rate (bilateral 2.3%, ipsilateral 0.5%, and no CLND 1%). They concluded that a contralateral neck dissection should only be performed in the presence of ipsilateral lymph nodes metastases on frozen section (24).

When assessing risks of CLND, it is important to consider that it is often more difficult to reoperate on patients with recurrent disease. In a study of reoperation for recurrent differentiated thyroid cancer, permanent vocal cord paralysis occurred in 18% of patients, mostly due to intentional nerve resection due to tumor invasion. In addition, hypoparathyroidism was also frequent in reoperative cases (29). A study by Shen et al. reviewed 295 CLNDs, 189 initial and 106 reoperations, comparing complications between the two surgery types (30). They found transient hypocalcemia was significantly higher in the initial CLND group (41.8 versus 23.6%), but there were no significant differences in permanent hypocalcemia, or transient or permanent hoarseness. Thus, they concluded that observing non-enlarged lymph nodes did not result in increased complications when reoperation was required. Recent retrospective studies have shown that observation of selected patients with low-risk PTC had low rates of recurrence, from 0.4 to 1.8% (31, 32), such that a small minority of patients will require a reoperation. As such, potential need for later reoperation should not, in and of itself, dictate the need for prophylactic CLND.

## Lateral Compartment Disease without Central Involvement

Generally, the most common pattern of lymph node spread is first to the central lymph node compartment (Level VI) followed by the lateral neck (Levels II–IV). In a retrospective review of patients who presented with clinically positive neck lymph nodes, 95% of patients had Level VI lymph node involvement, while the lateral neck was involved between 54 and 68% of the time (33). The pattern of spread suggests a typical step-wise progression from central to lateral neck compartments. As such, several groups propose that prophylactic removal of central neck nodes is beneficial in preventing local recurrence and further metastatic spread to regional lymph nodes. Nonetheless, there is the potential for “skip metastases” where patients have spread to lateral cervical nodes, without disease in the central neck. Reports of such phenomena have ranged from 6.8 to 37.5% for node-positive PTC (34–36). Studies have looked at predictors of “skip metastases,” showing higher likelihood in tumor in either the upper poles or lateral aspects of the thyroid lobe, as these tumors may be in closer proximity to nodes in the jugular chain than the more inferior central nodes. Although this brings into question the potential benefit of preventing further spread by clearing central neck micrometastases, it is important to note that “skip metastases” occur in the minority of patients. Still, when clinically positive lateral cervical lymph nodes are diagnosed preoperatively, standard-of-care remains to routinely perform a CLND at the time of resection. In addition, further studies have shown that if lymph node sampling is performed, a formal dissection should be performed rather than selective “berry-picking” of abnormal lymph nodes (37, 38).

## Patient Selection for Prophylactic Central Neck Lymph Node Dissection

Although there is strong consensus to perform CLND for therapeutic purposes, there is currently considerable controversy among endocrine surgeons regarding which patients should undergo prophylactic CLND for PTC. The 2009 ATA consensus statement recommends therapeutic CLND for any patients with clinically positive nodes and prophylactic CLND for patients with T3 and T4 primary tumors without evidence of nodal metastases, or with known lateral lymph node metastasis (39, 40). These general recommendations remained intact in the 2015 update, with the addition that prophylactic CLND may be performed if the information gained will guide further steps in therapy (41). As well, the 2015 guidelines add a statement that it is appropriate to not perform a prophylactic CLND for T1 or T2 tumors.

The controversies surrounding performance of a prophylactic CLND can be reflected in the varying recommendations from other national and international consensus groups as seen in Table 1. The National Comprehensive Cancer Network expert panel gives prophylactic CLND a Category 2B recommendation, stating that performance for patients with T3 or T4 tumors could be considered, but must be weighed against the increased risk of hypoparathyroidism and nerve injury (42). The British Thyroid Association, in their recommendations released in 2014, states that the benefit for prophylactic CLND in high-risk patients is unclear, and as such, they state that decision-making should be personalized. They do state that bilateral CLND has a benefit over ipsilateral CLND (43). The European Society of Endocrine Surgeons state that prophylactic CLND should be considered in those with high-risk features, including T3-4 tumors, age <15 or >45, male gender, bilateral or multifocal disease, or known lateral neck lymph node metastases (44). As well, this group emphasizes the importance of prophylactic CLND being done by surgeons in specialized centers. In contrast to these organizations, the Japanese Society of Thyroid Surgeons/Japanese Association of Endocrine Surgeons recommends routine performance of prophylactic CLND, based on increased risk of complications if surgery is needed for lymph node recurrence (45).

**Table 1:** Summary of recommendations from consensus groups regarding performance of prophylactic central neck lymph node dissection (CLND) for papillary thyroid cancer (PTC).

Consensus group	Year	Recommendations for prophylactic CLND for PTC
American Thyroid Association (41)	2015	Consider for T3/T4 tumors or clinically involved lateral neck nodes or if the information will impact further steps in therapy
National Comprehensive Cancer Network (42)	2016	Consider for patients with T3/T4 tumors, but must weight against the risk of hypoparathyroidism and nerve injury
British Thyroid Association (43)	2014	Benefit is unclear in high-risk patient, such that decision-making should be personalized. Bilateral CLND should be performed over ipsilateral CLND
European Society of Endocrine Surgeons (44)	2014	To be considered for patients with high-risk features, including T3/T4 tumors, extremes of age, male gender, bilateral/multifocal disease, clinically positive lateral lymph nodes. To be performed in specialized centers
Japanese Society of Thyroid Surgeons/Japanese Association of Endocrine Surgeons (45)	2011	To be performed routinely

High-risk features of the primary tumor might help predict nodal positivity; however, many of these features are unknown prior to surgery. Some factors that can be assessed preoperatively and are associated with aggressive disease include extremes of age, male gender, large primary tumor size, or bilateral disease. Pathologic features that are predictive of aggressive variants of PTC include tumor subtypes such as tall cell, columnar cell, Hurthle cell, diffuse sclerosis, and insular variants, as well as the presence of vascular invasion, extrathyroidal extension, and poorly differentiated tumors (46). Roh et al. prospectively examined 184 patients with unilateral PTC and clinically node negative disease by physical exam and ultrasound. All patients underwent total thyroidectomy and prophylactic CLND. The overall rates of lymph node metastasis were 42.9% to the ipsilateral central neck and 9.8% to the contralateral central neck compartments. In their multivariate analysis, tumor size >1 cm, extrathyroidal extension, and age <45 years were predictive of ipsilateral metastases. In addition, ipsilateral positive lymph nodes were most predictive of contralateral central neck positive lymph nodes. Of note, the rate of occult contralateral thyroid papillary microcarcinoma was 16.7% (5). In their review of 273 patients treated at the University of Chicago, Siddiqui et al. (47) found that age <45, multifocality, and extrathyroidal extension increased risk of central neck lymph node metastasis. They further systematically evaluated 10 previous studies that assessed risk factors for central neck lymph node metastases, including age, sex, tumor size, multifocality, bilaterality, thyroiditis, and extrathyroidal extension. There were no factors that were consistently associated with metastasis, yet the most common factors were large tumor size (eight studies) and presence of extrathyroidal extension (eight studies).

As in other cancer types, it has been proposed that sentinel lymph node biopsy could be performed to find patients with occult PTC lymph node metastases not identified by traditional preoperative and intraoperative methods. In these cases, if the sentinel node is positive, the patient would undergo a therapeutic CLND, and if the node is negative, the patient could be spared the increased morbidity of an unnecessary CLND. Several groups have shown promising initial results with traditional methods including methylene blue or isosulfan blue dye (48–50) and radioisotope injections, although many of these studies did not differentiate between central and lateral lymph nodes. These studies, as well as more recent studies assessing nanoparticles (51) and single-photon emission computed tomography and computed tomography (SPECT/CT) (52) show feasibility of technique, while many admit to high false negative rates. There is no direct evidence that sentinel lymph node biopsy has long-term benefits on patient outcomes (53). However, future studies of these more novel technologies may improve our ability to reliably select which patients should undergo CLND.

A greater understanding of molecular signaling pathways has the potential to better stratify patients who could benefit from prophylactic CLND. It is well known that mutations in the MAPK pathway are frequent driver mutations for PTC. One of the strongest activators of the MAPK pathway is the BRAF gene, found on chromosome 7. The most common genetic alteration found in PTC is the BRAF^V600E^ mutation, with a reported frequency of 40–70% (54, 55). In a retrospective review by Tufano et al., of 120 patients who underwent reoperative central neck dissection, 75% demonstrated the BRAF^V600E^ mutation (56). The patients with a BRAF mutation had a shorter time to recurrence, higher incidence of positive lateral lymph nodes, and a greater number of positive lymph nodes (56). Recently, it has become possible to test for BRAF mutations in preoperative FNA samples. One study showed 100% sensitivity and specificity for detecting the BRAF mutation in such specimens (57). Howell et al. found in a cohort of 156 patients that among the preoperative clinical parameters commonly used to determine if CLND is needed, only BRAF^V600E^ mutation was an independent predictor of metastases found in resected lymph nodes (58). However, additional recent studies, including a group of four institutions in the United States (59) as well as a group from South Korea (60) found the BRAF^V600E^ mutation was not an independent predictor of central neck lymph node metastases when looking specifically at conventional type PTC. Of note, the study from Han et al. did find that several microRNAs, including miR-146b-3p, miR-146-5p, and miR-222, were predictive of central neck lymph node metastases. Recently, combined BRAF^V600E^ and telomerase gene (TERT) promoter mutations have shown to have significantly shorter progression-free survival (61), clearly suggesting more aggressive tumor biology. As it stands currently in the published literature, there is no definite molecular marker, even BRAF^V600E^, which can definitely predict central neck lymph node metastases. As such, we would not recommend an established molecular test to guide operative decision-making. Nonetheless, as more factors are uncovered and their interrelationships elucidated, preoperative molecular testing may one day allow for stratifying patients who are candidates for prophylactic CLND. This provides a clear avenue of translating basic science discoveries into improvements in clinical care.

At our institution, there are no hard patient characteristics that define the use of prophylactic CLND, as different surgeons have different thresholds as to when it is appropriate. We always perform a thorough neck ultrasound performed by either the surgeon themselves or a referring endocrinologist skilled in ultrasound. Detailed attention is given to the characteristics of the primary tumor, such as local invasion, as well as the degree of suspicion in lymph nodes in the central and lateral cervical compartments with potential FNA of nodes as previously discussed. Certain characteristics about the primary tumor favor the use of prophylactic CLND. Extrathyroidal extension either seen on preoperative ultrasound or discovered intraoperatively warrants this procedure. Multifocal disease, if known preoperatively, favors it use as well. If lymph nodes are found to be slightly enlarged either on ultrasound or intraoperatively, a prophylactic CLND is more likely, even if the enlarged nodes have a benign appearance. Size of the primary tumor also impacts the use of prophylactic surgery. CLND is virtually never done for cancers <1 cm, but usually done for tumors >3 cm. A prophylactic CLND is more likely to be done on male patients and older patients given the worse prognosis with lymph node involvement. If there is concern about recurrent laryngeal nerve function at the time of surgery, then a prophylactic CLND is less likely to be done. In the future, we would hope that more precise molecular profiling could be performed on these patients preoperatively in order to more reliably assess the patients who would best benefit from prophylactic CLND.

## Conclusion

Papillary thyroid cancer is the most common thyroid malignancy, and metastatic spread involving lymph nodes of the central neck is common. The role of prophylactic CLND for PTC continues to be controversial. With the available evidence, we advocate a selective approach to performing prophylactic CLND. Per many international consensus panels, patients with larger tumors and unfavorable patient characteristics are more likely to benefit from prophylactic CLND, making these patients likely candidates for this procedure. However, such patients should also be aware of the potential for increased risks of hypoparathyroidism and recurrent nerve injury after a CLND over thyroidectomy, as well as questionable long-term benefits. Although recently tested molecular markers have contradictory findings, discovery of novel mutations and other biomarkers could potentially be used to better predict preoperatively that patients should undergo a CLND at the time of thyroidectomy in a more personalized manner.

## Author Contributions

JP provided the idea and critical review. NJ contributed writing parts of the initial text. LS wrote the completed text.

## Conflict of Interest Statement

The authors declare that the research was conducted in the absence of any commercial or financial relationships that could be construed as a potential conflict of interest.
